# Higher fat-soluble vitamin and phosphorus intake are associated with less dental caries among children and adolescents in the United States, NHANES 2011–2018

**DOI:** 10.3389/froh.2025.1617695

**Published:** 2025-07-09

**Authors:** Durdana Khan, Ixel Hernandez-Castro, Doreen Y. Larvie, Seth M. Armah, Andres Cardenas, Ashley J. Malin

**Affiliations:** ^1^ Department of Epidemiology, College of Public Health and Health Professions and College of Medicine, University of Florida, Gainesville, FL, United States; ^2^ Department of Epidemiology and Population Health, Stanford School of Medicine, Stanford, CA, United States; ^3^ Division of Nutritional Sciences, Cornell University, Ithaca, NY, United States; ^4^ Department of Nutrition, University of North Carolina Greensboro, Greensboro, NC, United States

**Keywords:** DMFT score, dental caries, dietary nutrients, fat soluble vitamins, phosphorus, NHANES, children, adolescents

## Abstract

**Introduction:**

Historic research shows that a diet rich in calcium, phosphorus, fat-soluble vitamins, and vitamin C, and low in phytates may help to prevent and arrest dental caries; however, current research on this topic is scarce. We examined associations of dietary intake of these nutrients with dental caries prevalence in the United States among youth 1–19 years old.

**Methods:**

The study included 2,676 young children (1–5 years), 3,214 older children (6–11 years) and 3,701 adolescents (12–19 years) from the National Health and Nutrition Examination Survey (NHANES 2011–2018). Daily nutrient intake was ascertained via two 24 h recalls. We assessed the number and presence (yes/no) of decayed and/or filled teeth (DFT) among young children and decayed, missing and/or filled teeth (DMFT) among older children and adolescents. Covariate-adjusted survey-weighted negative binomial regression was used to examine associations of nutrient quartiles with DFT or DMFT scores. We examined joint associations of nutrients with the probability of caries using the probit extension of Bayesian Kernel Machine Regression.

**Results:**

Mean (SD) DFT or DMFT scores were 0.82 (2.23) for young children, 2.08 (2.81) for older children and 2.51 (3.35) for adolescents. Higher phosphorus and vitamin A intake was associated with fewer DFT among young children [incident rate ratio (IRR) = 0.52, 95% CI: 0.29–0.94, *p* = 0.03, and IRR = 0.60, 95% CI: 0.37–0.95, *p* = 0.03, respectively]. Unexpectedly, higher intake of phytates was also associated with lower DFT scores among young children (IRR = 0.37, 95% CI: 0.21–0.65, *p* = 0.001). Higher phosphorus and vitamin E intake was associated with fewer DMFT among older children (IRR = 0.58, 95% CI: 0.40–0.84, *p* = 0.003 and IRR = 0.73, 95% CI: 0.54–0.97, *p* = 0.03, respectively). For adolescents, higher phosphorus and vitamin K intake was associated with fewer DMFT (IRR = 0.72, 95% CI: 0.53–0.99, *p* < 0.05; IRR = 0.82, 95% CI: 0.68–0.97, *p* = 0.02, respectively). The joint effect of nutrients was also associated with lower odds of DMFT. Setting all nutrients at their 75th relative to 50th percentiles was associated with 0.87 [95% credible interval (CrI): 0.81, 0.94] and 0.92 (95% CrI: 0.85, 0.99) lower odds of DMFT in older children and adolescents, respectively. Phosphorus and vitamin K contributed most to these associations.

**Conclusion:**

Fat-soluble vitamins and phosphorus may have systemic dental benefits that warrant further investigation.

## 1 Introduction

Dental caries, commonly known as tooth decay, remains a significant health concern among children and adolescents in the United States. Despite increased dental visits, the high prevalence of dental caries persists (1, 2). Approximately, 45.8% of youth aged 2–19 years were reported to be affected by dental caries from 2015–2017 (3); with 23% of children aged 2–5 years (4), 52% of children aged 6–8 years (4), and 57% of adolescents of ages 12–19 years affected (2, 4).

Current conceptualizations of the pathogenesis of dental caries suggest that it involves a dynamic interplay of demineralization and remineralization processes, influenced by factors such as bacterial activity, dietary sugars, saliva composition, and fluoride exposure (5). Biofilms, which are complex aggregations of microorganisms adhering to dental surfaces, are integral to the pathogenesis of dental caries. The extracellular matrix of these biofilms can provide a protective niche for resident bacteria that contribute to dental caries. Acidogenic bacteria, particularly mutans streptococci, flourish within these biofilms, significantly contributing to the progression of caries (6–8). However, biofilms can also serve as a reservoir for fluoride, or even alkaline microorganisms, that can help to remineralize teeth, as well as prevent harmful bacteria from contributing to infections (9–12). These localized effects are well-documented, and form the basis for many preventive strategies including reducing sugar intake, use of fluoridated dental products, and the maintenance of optimal oral hygiene practices (2).

While most literature focuses on local dietary effects in the oral cavity (13–18); the potential systemic influence of nutrition on caries formation remains underexplored (19). Better understanding how the diet could potentially systemically influence dental caries progression, may be a missing piece to addressing this prevalent public health issue. While there are few recent studies on this topic, historic animal and human studies (conducted primarily in children or adolescents) published in the early to mid-1900s suggest that diets rich in calcium, phosphorus, vitamin C, and fat-soluble vitamins (A, D, and K2), with low phytate content, may help to prevent and even reverse dental caries by providing essential nutrients to the structure of teeth that help to systemically remineralize them [see (20) for a review]. However, this hypothesis has not yet been explored in the contemporary literature. Therefore, the present study examined associations of dietary calcium, phosphorus, vitamin C, fat-soluble vitamins (A, D, K, and E) and phytate intake with dental caries prevalence in a nationally representative sample of United States youth participating in the National Health and Nutrition Examination Survey (NHANES). We hypothesized that diets higher in calcium, phosphorus, vitamins A, D, K, E, and C and lower in phytates would be inversely associated with caries prevalence among youth.

## 2 Materials and methods

### 2.1 Participants

**Figure 1:**
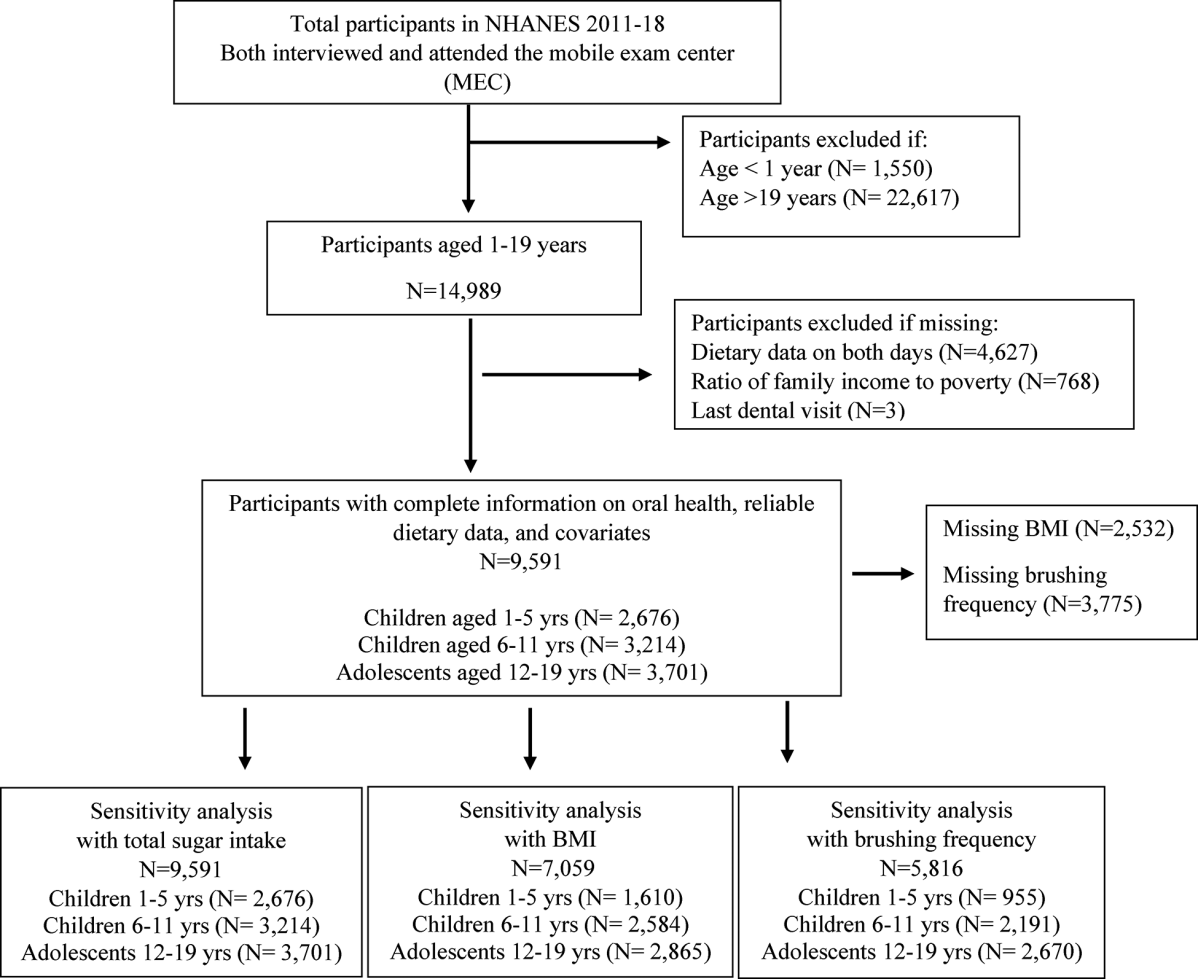
Flow chart of study participants included in the final analysis.

This study included participants from cycles 2011–2018 of NHANES (see **eMethods** for NHANES survey procedures) (21). There were 39,156 participants who completed the interview portion of NHANES 2011–2018. Of these, a subsample of 9,591 participants aged 1–19 years received an oral health examination, had reliable dietary data, and had complete data on all covariates included in the analysis. The final sample in the present analysis was comprised of 2,676 children aged 1–5 years, 3,214 children aged 6–11 years, and 3,701 adolescents aged 12–19 years. See Figure 1 for a participant selection flow diagram.

### 2.2 Measures

#### 2.2.1 Dietary intake

Daily dietary intake data were obtained from two 24-hour dietary recalls known as “What We Eat in America” (WWEIA), the dietary intake component of NHANES. These were carried out through a collaboration between the U.S. Department of Agriculture (USDA) and the U.S. Department of Health and Human Services (DHHS) (22, 23). The first dietary recall was obtained during an initial in-person interview and the second was obtained 3–10 days later via telephone (23, 24). Interviews were conducted for participants less than six years of age with a proxy person (generally the person most knowledgeable about the participant's intake); interviews of children aged 6–11 years were conducted with a proxy person and the child present (24). Participants 12 years or older answered for themselves. We only included participants whose 24 h dietary recalls were considered reliable as assessed based on standard NHANES criteria of completing all relevant variables associated with the 24 h dietary recall. We included dietary recall data from both days (23).

#### 2.2.2 Nutrient intake calculations

We calculated the mean dietary intake of calcium, phosphorus, vitamin C, fat-soluble vitamins (A, D, K, and E) from two days of recall, employing a widely recognized methodology (25). By utilizing this approach (two days of recall), we accounted for the variability in dietary nutrient consumption, thereby providing a more accurate representation of participants' habitual dietary patterns. However, we derived phytate intake from only the first day due to phytate data not being available for the second day at the time of analysis.

Phytate intake was estimated using the method published by Larvie and Armah (26). This method uses the USDA Food Patterns Equivalents Database (FPED) and phytate content values from the Food and Agriculture Organization/International Network of Food Data Systems/International Zinc Nutrition Consultative Group (FAO/INFOODS/IZINCG) Global Food Composition Database for Phytate (27, 28). The FPED converts food and beverages reported in WWEIA into 37 food pattern components/food group (29). We used data for the 2011–2018 survey cycles (30) and focused on food groups that contain phytate (e.g., dark green vegetables, other starchy vegetables, legumes, whole grains, refined grains, soy products, nuts and seeds).

To estimate the phytate intake, first, the number of equivalents of each food group consumed by each participant was converted to weight in grams using weight per unit equivalents data obtained from the FPED Methodology and User Guide document (29). Since the FPED document provides weight per unit equivalent data for individual food items, we estimated the values for each food group by averaging the weights provided in the FPED document for frequently consumed foods under that food group. For instance, for dark green leafy vegetables, we averaged the weight/unit equivalents for beet greens, broccoli, Chinese cabbage, collards, spinach, and turnip. The FAO/INFOODS/IZINCG phytate content data was then applied to the FPED data to estimate the phytate intake from each of the different food groups by each participant per day. The FAO/INFOODS/IZINCG data provides phytate content in mg per 100 g at the food group level, thus making it possible to estimate how much phytate was consumed from each food group for each participant. To obtain the total daily phytate intake in mg for each participant, we summed up the phytate intake from all the different food groups consumed. The data were then merged with the corresponding 2011–2018 NHANES data.

#### 2.2.3 Dental caries

Oral health examinations were conducted by licensed dentists in a room at the MEC using light, compressed air, and a portable dental chair. Tooth scoring criteria used in the dental examination, along with quality assurance and training/calibration details, are further described in the NHANES plan and operations manual (31, 32).

##### 2.2.3.1 Calculation of decayed, missing, and filled teeth (DMFT) and decayed and filled teeth (DFT) scores

The DMFT index, representing decayed, missing, and filled teeth, is a widely used measure (33) in NHANES to assess dental health. DMFT was calculated as the total count of decayed, missing, or filled status of each tooth. NHANES dental codes are summarized in Supplementary Table S1. A tooth was denoted “decayed” if it had an untreated carious lesion or both a carious lesion and a restoration (32). NHANES employed precise diagnostic criteria (visual and tactile criteria) to assess and document coronal caries, encompassing both frank lesions (gross cavitation) and incipient lesions (the early stages of caries development prior to cavity formation) (see eMethods) (32). Teeth missing due to caries were recorded as “missing.” Teeth with temporary or permanent fillings/restoration were classified as “filled.” Teeth that were congenitally missing, unerupted, supernumerary teeth, third molars, and teeth missing for reasons other than dental causes were excluded from DMFT scoring. The DMFT index was considered for permanent teeth, which are relevant for individuals beyond the age of 6 when primary dentition transitions to permanent dentition (34, 35). For children aged 1–5 years, we calculated a DFT score, which represents decayed and filled teeth, capturing both treated and untreated caries in primary dentitions. We excluded the “missing” component for this age group because primary teeth naturally fall out as part of the child's development, making it difficult to distinguish between teeth missing due to caries and those missing due to natural exfoliation (35). For older children (6–11 years) both primary and permanent dentitions were included in creating DMFT scores depending on what type of teeth the participant had. To avoid duplication, only the permanent tooth was scored when both primary and permanent teeth were present in the same space. If both a permanent and a primary tooth were visible in the same tooth space, only the status of the permanent tooth was described and no score was assigned for the primary tooth (32). For all participants we calculated a count indicator measuring the total number of DFT or DMFT, as well as a binary (yes/no) indicator measuring whether any DFT or DMFT were present.

### 2.3 Covariates

We included sociodemographic variables that have been associated with dental caries among children and adolescents, as well as with dietary nutrient intake in previous studies (36–38). These include age, sex, race/ethnicity, the ratio of family income to poverty, last dental visit and total energy intake. Race/ethnicity was classified as Mexican American, Other Hispanic, non-Hispanic White, non-Hispanic Black, non-Hispanic Asian, and Other Race, Including Multiracial; sex was defined as male or female; the ratio of family income to poverty was calculated by dividing annual family income by the poverty guidelines for the survey year. The Department of Health and Human Services (HHS) poverty guidelines were used as the poverty measure to calculate the family income to poverty ratio (39). The values range from 0–5 and were not computed if family income data were missing. Total energy intake was defined as total kcal per day and last dental visit was re-categorized as 6 months to 1 year, more than 1 year to 3 years, more than 3 years or never have been, and refused/don't know. Participants were stratified into age groups of 1–5 years, 6–11 years and 12–19 years for statistical analyses.

### 2.4 Statistical analysis

Descriptive statistics were reported for participant demographic characteristics, dietary nutrient intakes and dental caries scores across different age groups. To address count data and account for over-dispersion, we employed covariate-adjusted negative binomial regression to examine associations of dietary nutrient intake with DFT or DMFT scores. This method indicated good model fit (see **eMethods**). We examined the association between quartiles of each dietary nutrient separately with DFT and DMFT scores and stratified models by age group. Coefficients were exponentiated and presented as incident rate ratios (IRR) with respective 95% confidence intervals and *p*-values. We also conducted supplemental covariate-adjusted logistic regression analyses to examine associations between quartiles of each dietary nutrient intake separately in relation to binary presence of DMFT or DFT (yes/no) stratified by age group. Exponentiated coefficients from these models were presented as odds ratios (OR) with their respective 95% confidence intervals and *p*-values. All these analyses applied sampling weights from two days of dietary recall to ensure representation of the US population. However, only sampling weights from day 1 were applied for phytate intake as it was exclusively derived from the first day of dietary recall.

To examine joint associations of nutrients as a mixture with the probability of DFT or DMFT, we applied the probit extension of Bayesian Kernel Machine Regression (BKMR). BKMR is a semi-parametric method employing a flexible Gaussian kernel function (40) that allows for non-linear exposure-response functions. Furthermore, it can evaluate potential synergistic and antagonistic relationships between exposures (41). Relative to traditional regression approaches, BKMR limits potential Type I error by reducing the number of models and multiple comparison issues. It is also particularly suitable for nutrient mixtures where moderate to high correlations between exposures may lead to collinearity in alternative approaches (41). Using probit-BKMR with a component-wise variable selection, we modeled the overall effect of concurrently increasing percentiles of all nutrients in the mixture on odds of DFT/DMFT vs. no DFT/DMFT. We also evaluated posterior inclusion probabilities of each individual nutrient to assess the relative importance of the nutrient to the overall mixture effect. We assessed the single-exposure health effects for each nutrient (univariate associations) with the odds of DFT or DMFT, as well as pairwise interactions between nutrients in relation to the odds of DFT or DMFT (see eMethods). All BKMR models were unweighted and reported odds ratios (OR) with their respective 95% credible intervals (CrI) (see eMethods for BKMR data preparation).

In both weighted negative binomial regression models and unweighted BKMR models we explored potential interactions between vitamin A and vitamin K, vitamin D and vitamin A, vitamin K and vitamin D, vitamin K and phosphorous, and vitamin C and vitamin K. All models were adjusted for age, sex, race/ethnicity, ratio of family income to poverty, total energy intake, and last dental visit. We performed sensitivity analyses adjusting for sugar intake (gm), body mass index (BMI) in kg/m^2^, and brushing frequency (<=2 times/day or >2 times/day) in negative binomial regression models and adjusting for sugar intake in BKMR models. Mixture BKMR models did not include the survey weights or design due to software limitations. All data management and negative binomial regressions were performed using STATA version 18.0 and BKMR analysis were run in R (R v.4.3) using the “*bkmr*” package.

## 3 Results

Descriptive statistics for participant demographic characteristics and dental caries measures are summarized in Table 1. The Mean (SD) age in years was 3.02 (1.38) for younger children, 8.52 (1.69) for older children, and 15.51 (2.27) for adolescents. The distribution of females and males was approximately equal among all age groups. Most participants identified as Non-Hispanic White and reported having had at least one dental visit within the last year. The prevalence of DFT in primary teeth among younger children aged 1–5 years was 17.43 (%). For older children aged 6–11 years, 50.65 (%) had DMFT, while for adolescents, 56.25 (%) had DMFT. The mean (SD) number of teeth with caries (i.e., DFT or DMFT score) was 0.82 (2.23) for children aged 1–5 years, 2.08 (2.81) for children aged 6–11 years, and 2.51 (3.35) for adolescents.

**Table 1:** Demographic characteristics of the study participants across different age groups.

Variable	Children	Children	Adolescents
	(1–5 years)	(6–11 years)	(12–19 years)
Total			
*n* = Unweighted	*n* = 2,676	*n* = 3,214	*n* = 3,701
*N* = Weighted	*N* = 17,994,454	*N* = 23,324,491	*N* = 31,371,034
Socio-demographic and behavioral variables			
Age; Mean (SD)	3.02 (1.38)	8.52 (1.69)	15.51 (2.27)
Sex; Freq (%)			
Male	9,259,150 (51.45)	11,957,976 (51.27)	15,942,678 (50.82)
Female	8,735,304 (48.54)	11,366,515 (48.73)	15,428,356 (49.18)
Race/Ethnicity; Freq (%)			
Mexican American	3,052,704 (17.96)	3,620,309 (15.52)	5,079,272 (16.19)
Other Hispanic	1,409,915 (7.83)	1,827,406 (7.83)	1,997,391 (6.37)
Non-Hispanic White	9,262,863 (51.47)	12,248,749 (52.51)	16,670,546 (53.14)
Non-Hispanic Black	2,324,403 (12.92)	3,147,010 (13.49)	4,531,542 (14.44)
Non-Hispanic Asian	784,471 (4.36)	989,506 (4.24)	1,450,449 (4.62)
Other race/multi-racial	1,160,097 (6.45)	1,491,510 (6.39)	1,641,832 (5.23)
Body Mass Index (BMI kg/m^2)^ (*n* = 7,059)	16.42 (1.82)	18.49 (4.06)	24.29 (6.31)
Ratio of family income to poverty; Mean (SD)	2.26 (1.63)	2.39 (1.61)	2.43 (1.64)
Dental behavior variables			
Last dental visit; Freq (%)			
6 months to 1 year	10,270,090 (57.07)	20,137,263 (86.33)	25,103,560 (80.02)
More than 1 year to 3 years	850,055 (4.72)	2,426,770 (10.40)	4,391,306 (13.99)
More than 3 years/Never have been	6,833,698 (37.97)	736,721 (3.16)	1,775,001 (5.66)
Refused/don't know	40,610 (0.22)	23,736 (0.10)	101,167 (0.32)
Frequency of brushing (*n* = 5,816)			
2 or less times per day	7,325,811 (94.05)	16,358,304 (97.06)	21,374,278 (93.64)
More than 2 times per day	463,016 (5.94)	494,017 (2.93)	1,449,358 (6.35)
Total energy (Kcal); Mean (SD)	1,453.47 (445.03)	1,903.57 (547.61)	1,991.53 (770.01)
Total sugar (gm); Mean (SD)	95.43 (37.09)	115.17 (43.94)	113.21 (58.37)
Dental outcome variables			
DFT score for primary teeth; Mean (SD)	0.82 (2.23)	–	–
DMFT score for permanent teeth; Mean (SD)	–	2.08 (2.81)	2.51 (3.35)
Prevalence of DMFT; Freq (%)			
No	–	11,509,667 (49.34)	13,722,941 (43.74)
Yes	–	11,814,824 (50.65)	17,648,094 (56.25)
Prevalence of DFT; Freq (%)			
No	14,857,114 (82.56)	–	–
Yes	3,137,340 (17.43)	–	–

IQR, inter quartile range; SD, standard deviation; Kcal, kilo calorie; DFT, decayed, filled teeth; DMFT, decayed, missing, filled teeth.

All estimates, including median, IQR, mean, SD, frequencies and column percentages were calculated using the 2 days dietary sampling weights to account for the complex survey design of NHANES.

Table 2 summarizes descriptive statistics for participants' daily dietary nutrient intakes according to recommended dietary allowance (RDA) per age group (42). On average, participants had adequate daily nutrient intake for most nutrients. However, median (IQR) intake of Vitamin D was lower than the RDA of 15 mcg among all age groups (42). Similarly, median (IQR) vitamin E intake was lower than the RDAs among all age groups, and median (IQR) calcium intake was lower than the RDAs for older children and adolescents (42, 43).

**Table 2:** Summary statistics and recommended levels of dietary nutrients according to age group.

Dietary Nutrient	Mean (SD)	Median (IQR)	Minimum	Maximum	Recommended Dietary Allowance (RDA)^a^
Children (1–5 years), *n* = 2,676					
Calcium (mg)	929.6 (397.3)	880.5 (494.5)	92.5	4,607.5	Age 1–3: 700 mg
					Age 4–8: 1,000 mg
Phosphorus (mg)	1,033.9 (447.9)	991.5 (443.5)	165	3,234.5	Age 1–3: 460 mg
					Age 4–8: 500 mg
Vitamin A (mcg)	545.2 (281.7)	504.7 (324.2)	17	4,120.5	Age 1–3: 300 mcg
					Age 4–8: 400 mcg
Vitamin C (mg)	79.5 (59.2)	66.3 (64.9)	0.55	906.7	Age 1–3: 15 mg
					Age 4–8: 25 mg
Vitamin D (mcg)	6.2 (3.6)	5.7 (4.2)	0	41.4	Age 1–13: 15 mcg
Vitamin E (mg)	5.4 (2.8)	4.8 (3.1)	0.5	34	Age 1–3: 6 mg
					Age 4–8: 7 mg
Vitamin K (mcg)	50.2 (44)	38.6 (32.6)	3	626.5	Age 1–3: 30 mcg^b^
					Age 4–8: 55 mcg^b^
Phytate (mg)	490.5 (349.9)	403 (370.5)	0	3,521.4	NA
Children (6–11 years), *n* = 3,214					
Calcium (mg)	1,215.6 (430.9)	952 (526.5)	83	4,418	Age 4–8: 1,000 mg
					Age 9–13: 1,300 mg
Phosphorus (mg)	1,259.6 (417.4)	1,217.5 (526.5)	153	4,245.5	Age 4–8: 500 mg
					Age 9–13: 1,250 mg
Vitamin A (mcg)	621.1 (356.5)	565 (388)	8.5	7,773.5	Age 4–8: 400 mcg
					Age 9–13: 600 mcg
Vitamin C (mg)	78.8 (56.8)	67.8 (68.7)	0.7	659.8	Age 4–8: 25 mg
					Age 9–13: 45 mg
Vitamin D (mcg)	5.6 (3.4)	5.1 (4)	0	46.3	Age 1–13: 15 mcg
Vitamin E (mg)	7.1 (3.3)	6.4 (3.8)	1.1	35.45	Age 4–8: 7 mg
					Age 9–13: 11 mg
Vitamin K (mcg)	69.2 (65)	52.3 (45.7)	4.15	1,180.1	Age 4–8: 55 mcg^b^
					Age 9–13: 60 mcg^b^
Phytate (mg)	679.6 (431.3)	580.1 (449.7)	0	5,371.8	NA
Adolescents (12–19 years), *n* = 3,701					
Calcium (mg)	958.4 (510.2)	869 (623.5)	31.5	5,237.5	Age 9–13: 1,300 mg
					Age 14–18: 1,300 mg
Phosphorus (mg)	1,274.4 (561.4)	1,186.5 (667)	63	5,535.5	Age 9–13: 1,250 mg
					Age 14–18: 1,250 mg
Vitamin A (mcg)	552.5 (384.3)	471 (428.5)	1	5,337.5	Age 9–13: 600 mcg
					Age 14–18: M: 900 mcg, F: 700 mcg
Vitamin C (mg)	71 (68.1)	53 (72.7)	0	1,635.8	Age 9–13: 45 mg
					Age 14–18: M: 75 mg, F: 65 mg
Vitamin D (mcg)	4.7 (4.3)	3.7 (4.4)	0	47.3	Age 14–18: 15 mcg
Vitamin E (mg)	7.5 (5.2)	6.5 (4.6)	0.4	115	Age 9–13: 11 mg
					Age 14 plus: 15 mg
Vitamin K (mcg)	75.6 (83.7)	54.8 (54.4)	0.95	1,828.7	Age 9–13: 60 mcg^b^
					Age 14–18: 75 mcg^b^
Phytate (mg)	710.3 (575.3)	576.6 (527.8)	0	7,641.1	NA

All nutrients are average intake values of day 1 and day 2, except for phytate which is calculated for day 1 only.

All estimates are unweighted.

RDAs and AIs are available at National Institutes of Health. Office of Dietary Supplements. Dietary Supplement Fact Sheets [Internet]. National Institutes of Health. [cited 2024 Mar 22]. Available from: https://ods.od.nih.gov/factsheets/list-all/.

^a^ Recommended Dietary Allowance (RDA): Average daily level of intake sufficient to meet the nutrient requirements of nearly all (97%–98%) healthy individuals.

^b^ Adequate Intake (AI): Intake at this level is assumed to ensure nutritional adequacy; established when evidence is insufficient to develop an RDA.

### 3.1 Associations between dietary nutrient intake and number of dental caries

Associations between individual dietary nutrient intakes and DFT or DMFT scores are presented in Table 3. Among younger children, higher intake of phosphorus was associated with lower DFT scores (IRR_Q2_ = 0.52, 95% CI: 0.35–0.76, *p* = 0.001; IRR_Q3_ = 0.52, 95% CI: 0.29–0.94, *p* = 0.030). Similarly, among older children and adolescents, higher intake of phosphorus was associated with lower DMFT scores [(IRR_Q2_ = 0.77, 95% CI; 0.61–0.97, *p* = 0.025; IRR_Q4_ = 0.58, 95% CI; 0.40–0.84, *p* = 0.003 for older children) and (IRR_Q3_ = 0.79, 95% CI; 0.62–1.00, *p* = 0.050; IRR_Q4_ = 0.72, 95% CI; 0.53–0.99, *p* = 0.045 for adolescents)]. However, when adjusting for BMI in a reduced sample, the association between higher phosphorous and lower DMFT scores was no longer statistically significant for adolescents (in Supplementary Table S2).

**Table 3:** Associations between quartiles of nutrients and dental decay across different age groups.

Dietary Nutrient Quartiles	DFT score for primary teeth (No. of decayed or filled teeth)		DMFT score for primary and permanent teeth (No. of decayed, missing or filled teeth)			
	Children Aged 1–5 years (*n* = 2,676)		Children Aged 6–11 years (*n* = 3,214)		Adolescents Aged 12–19 years (*n* = 3,701)	
	Incident Rate Ratios	*p*-value	Incident Rate Ratios	*p*-value	Incident Rate Ratios	*p*-value
	IRR (95% CI)		IRR (95% CI)		IRR (95% CI)	
Calcium (mg)						
Q1 (31.5–594)	Reference		Reference		Reference	
Q2 (595.5–842)	0.71 (0.44–1.15)	0.169	0.89 (0.69–1.16)	0.400	0.97 (0.80–1.17)	0.720
Q3 (842.5–1,148.5)	0.79 (0.49–1.27)	0.324	0.85 (0.66–1.09)	0.200	0.93 (0.77–1.13)	0.482
Q4 (1,149–5,237.5)	0.74 (0.41–1.32)	0.303	0. 87 (0.65–1.16)	0.349	0.85 (0.68–1.06)	0.114
Phosphorus (mg)						
Q1 (63–888)	Reference		Reference		Reference	
Q2 (888.5–1,180.5)	0.52 (0.35–0.76)	**0**.**001**	0.77 (0.61–0.97)	**0**.**025**	0.89 (0.73–1.09)	0.270
Q3 (1,181–1,531.5)	0.52 (0.29–0.94)	**0**.**030**	0.83 (0.64–1.08)	0.164	0.79 (0.62–1.00)	**0**.**050**
Q4 (1,532.5–5,535.5)	0.55 (0.25–1.20)	0.135	0.58 (0.40–0.84)	**0**.**003**	0.72 (0.53–0.99)	**0**.**045**
Vitamin A (mcg)						
Q1 (1–327.5)	Reference		Reference		Reference	
Q2 (328–511.5)	0.68 (0.42–1.09)	0.111	0.92 (0.74–1.14)	0.437	0.92 (0.77–1.10)	0.357
Q3 (512–754.5)	0.60 (0.37–0.95)	**0**.**031**	1.01 (0.80–1.28)	0.925	0.88 (0.73–1.07)	0.198
Q4 (755–7,773.5)	0.64 (0.34–1.21)	0.166	0.95 (0.72–1.25)	0.733	0.91 (0.74–1.11)	0.359
Vitamin C (mg)						
Q1 (0–31.4)	Reference		Reference		Reference	
Q2 (31.45–63.15)	1.23 (0.72–2.11)	0.440	0.84 (0.66–1.07)	0.166	0.97 (0.81–1.17)	0.789
Q3 (63.2–108.05)	1.25 (0.72–2.16)	0.430	1.03 (0.81–1.31)	0.806	0.88 (0.73–1.07)	0.202
Q4 (108–1,635.85)	1.95 (1.05–3.60)	**0**.**033**	0.83 (0.66–1.05)	0.129	0.88 (0.72–1.07)	0.191
Vitamin D (mcg)						
Q1 (0–2.1)	Reference		Reference		Reference	
Q2 (2.15–4)	0.98 (0.51–1.87)	0.946	1.16 (0.89–1.51)	0.265	1.24 (1.05–1.48)	**0**.**013**
Q3 (4.05–6.7)	0.83 (0.48–1.43)	0.498	0.96 (0.74–1.26)	0.791	1.03 (0.86–1.24)	0.726
Q4 (6.75–47.3)	0.76 (0.42–1.40)	0.380	1.02 (0.77–1.37)	0.844	1.04 (0.85–1.27)	0.680
Vitamin E (mg)						
Q1 (0.4–4.71)	Reference		Reference		Reference	
Q2 (4.72–6.74)	1.07 (0.72–1.57)	0.749	0.79 (0.64–0.98)	**0**.**034**	1.06 (0.87–1.29)	0.587
Q3 (6.75–9.59)	1.08 (0.67–1.73)	0.747	0.79 (0.62–1.00)	**0**.**050**	0.97 (0.79–1.20)	0.814
Q4 (9.6–115.03)	1.31 (0.70–2.46)	0.395	0.73 (0.54–0.97)	**0**.**031**	1.02 (0.81–1.29)	0.861
Vitamin K (mcg)						
Q1 (0.95–39.65)	Reference		Reference		Reference	
Q2 (39.7–64.65)	0.89 (0.64–1.26)	0.529	0.85 (0.69–1.04)	0.123	0.82 (0.68–0.97)	**0**.**024**
Q3 (64.7–111.2)	1.65 (0.93–2.94)	0.089	0.83 (0.66–1.04)	0.107	0.82 (0.67–1.00)	**0**.**052**
Q4 (111.25–1,828.65)	1.51 (0.80–2.86)	0.200	0.89 (0.68–1.15)	0.366	0.85 (0.69–1.05)	0.142
Phytate (mg)						
Q1 (0–342.43)	Reference		Reference		Reference	
Q2 (342.53–566.41)	0.68 (0.44–1.06)	0.092	1.02 (0.82–1.25)	0.886	0.98 (0.80–1.20)	0.850
Q3 (566.62–908.66)	0.47 (0.31–0.72)	**<0**.**001**	0.87 (0.70–1.08)	0.212	0.95 (0.78–1.17)	0.652
Q4 (908.71–7,641.07)	0.37 (0.21–0.65)	**0**.**001**	0.84 (0.66–1.06)	0.144	0.85 (0.63–1.07)	0.166

The estimates, 95% CIs and *p*-values were calculated using the 2 days dietary sampling weights to account for the complex survey design of NHANES.

For models with phytates the day 1 dietary weights were used.

These negative binomial regression models are adjusted for age, sex, race/ethnicity, ratio of family income to poverty, total energy intake, and last dental visits.

Bold values represent significant *p*-value < 0.05.

Higher dietary intake of fat-soluble vitamins was also associated with less dental caries across all age groups; notably, vitamins A, E, and K showed inverse associations. For younger children, a higher intake of vitamin A was associated with lower DFT scores (IRR_Q3_ = 0.60, 95% CI: 0.37–0.95, *p* = 0.031); for older children, higher intake of vitamin E was associated with lower DMFT scores (IRR_Q2_ = 0.79, 95% CI: 0.64–0.98, *p* = 0.034; IRR_Q3_ = 0.79, 95% CI: 0.62–1.00, *p* = 0.05; IRR_Q4_ = 0.73, 95% CI: 0.54–0.97, *p* = 0.031); and for adolescents, higher vitamin K intake was associated with lower DMFT scores (IRR_Q2_ = 0.82, 95% CI: 0.68–0.97, *p* = 0.024; IRR_Q3_ = 0.82, 95% CI: 0.67–1.00, *p* = 0.052). Interestingly, when adjusting for brushing frequency, higher vitamin K intake was associated with lower DMFT scores among older children but not adolescents, yet all other findings remained consistent (see Supplementary Table S3).

Unexpectedly, higher intake of phytates was associated with lower DFT scores among younger children (IRR_Q3_ = 0.47, 95% CI: 0.31–0.72, *p* < 0.001; IRR_Q4_ = 0.37, 95% CI: 0.21–0.65, *p* = 0.001). In contrast, the highest intake of vitamin C was associated with higher DFT scores among younger children (IRR_Q4_ = 1.95, 95% CI: 1.05–3.60, *p* = 0.033). However, this association might be confounded by sugar intake because it became non-significant after adjusting for daily sugar intake in sensitivity analyses (see Supplementary Table S4). Furthermore, for older children, after adjusting for BMI, higher vitamin C intake was associated with lower DMFT scores (IRR_Q2_ = 0.75, 95% CI: 0.57–0.98, *p* = 0.033). None of the interactions tested were statistically significant.

In sensitivity analyses, when we examined nutrient intake in relation to odds of dental caries (yes/no), findings remained generally consistent. However, higher intake of vitamin E and vitamin K were associated with higher odds of DFT in young children. Nevertheless, for older children and adolescents, higher intakes of these nutrients continued to be associated with lower odds of DMFT (see Supplementary Table S5).

### 3.2 Associations of nutrient mixtures with presence of DFT or DMFT

**Figure 2:**
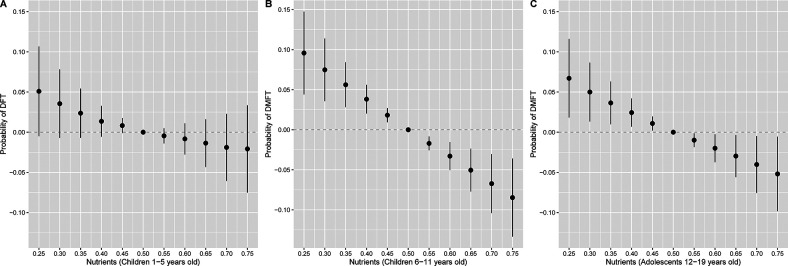
Overall association of the nutrient mixture on the probability of **(A)** DFT (*n* = 2,676) in children 1–5 years old or **(B)** DMFT in children 6–11 years old (*n* = 3,214) and **(C)** 12–19 years old (*n* = 3,701). Figures illustrate the probit model estimates and 95% credible intervals for DFT or DMFT vs. no DFT or DMFT of nutrients held at the percentile specified on the x-axis when compared with setting nutrients to their median values. Models were adjusted for age, sex, race and ethnicity, ratio of family income to poverty, total energy, and last dental visit.

The joint association between nutrients and DFT in children 1–5 years old or DMFT in children 6–11 and adolescents is depicted in Figure 2. Higher intake of nutrients as a mixture was associated with lower odds of DMFT in children (6–11 years) and adolescents (12–19 years). Holding all nutrients at their 75^h^ percentile relative to 50th percentile was associated with a 0.87 (95% CrI: 0.81, 0.94) and 0.92 (95% CrI: 0.85, 0.99) lower odds of DMFT compared to no DMFT in children 6–11 and 12–19 years old, respectively (Table 4). A similar inverse pattern was observed between the nutrient mixture and DFT in children 1–5 years old, however, the 95% CrI crossed the null. Dietary phosphorous and vitamin K intake were identified as the most important contributors to the nutrient mixture in DMFT models for children 6–11 and 12–19 years old (Table 4). In univariate exposure-response plots for DMFT in children 6–11 years old (Supplementary Figure S1), phosphorous was also inversely associated with DMFT, with lower probability of DMFT at higher concentrations of phosphorous, holding all other nutrients at their median. Univariate exposure-response associations for DMFT in children 12–19 years old with vitamin K were slightly u-shaped, with higher probability of DMFT at the lowest and highest concentrations of vitamin K, when holding other nutrients at their median. We did not find evidence of pairwise interactions between nutrients in relation to DFT or DMFT in children (Supplementary Figures S2, S3). However, a pairwise interaction was visually observed between vitamin K and vitamin C in the DMFT model for adolescents (Supplementary Figure S4). Nevertheless, we did not find significant interactions between vitamin K and vitamin C in the negative binomial regression models.

**Table 4:** BKMR results for the covariate adjusted joint effects of the nutrient mixture on odds of DFT or DMFT .

Nutrient Mixture	Children Age 1–5 years	Children Age 6–11 years	Adolescents Aged 12–19 years
	(*n* = 2,676)	(*n* = 3,214)	(*n* = 3,701)
Posterior Inclusion Probabilities (PIPs)^a^			
Calcium	0.05	0.33	0.32
Phosphorous	0.78	0.84	0.59
Vitamin A	0.14	0.24	0.22
Vitamin C	0.84	0.23	0.39
Vitamin D	0.10	0.26	0.16
Vitamin E	0.08	0.45	0.22
Vitamin K	0.15	0.32	0.85
Phytate	0.37	0.19	0.11
Overall Mixture Effect Estimates^b^	OR (95% CI)	OR (95% CI)	OR (95% CI)
75th vs. 50th percentile	0.97 (0.89, 1.06)	0.87 (0.81, 0.94)	0.92 (0.85, 0.99)

Models were adjusted for age, sex, race and ethnicity, ratio of family income to poverty, total energy, and last dental visit.

BKMR, Bayesian kernal machine regression.

^a^ Posterior inclusion probabilities represent the relative importance of each nutrient to the overall mixture effect.

^b^ Odds ratio for DFT or DMFT when concentrations of all nutrients are held at their 75th percentile compared to when they are all held at the 50th percentile.

**Figure 3:**
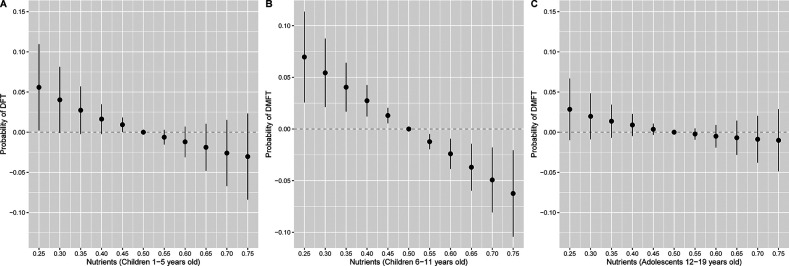
Overall association of the nutrient mixture on the probability of **(A)** DFT (*n* = 2,676) in children 1–5 years old or **(B)** DMFT in children 6–11 years old (*n* = 3,214) and **(C)** 12–19 years old (*n* = 3,701), additionally adjusting for daily sugar intake. Figures illustrate the probit model estimates and 95% credible intervals for DFT or DMFT vs. no DFT or DMFT of nutrients held at the percentile specified on the x-axis when compared with setting nutrients to their median values. Models were adjusted for age, sex, race and ethnicity, ratio of family income to poverty, total energy, last dental visit, and daily sugar intake.

We observed single exposure health effects for phosphorous and Vitamin C in DFT mixtures models for children 1–5 years old (Supplementary Figure S5). When holding all other nutrients at their median, individual changes in phosphorous from its 25th to 75th percentile were associated with 0.75 (95% CrI: 0.63, 0.89) lower odds of DFT, while individual changes in Vitamin C from its 25th to 75th percentile were associated with 1.25 (95% CrI: 1.13, 1.38) higher odds of DFT (Supplementary Table S6). In DMFT mixtures models for children 6–11 years old, we found single exposure effects for phosphorous (Supplementary Figure S5) such that changes from the 25th to 75th percentile were associated with 0.79 (95% CrI: 0.67, 0.94) lower odds of DMFT (Supplementary Table S6). For DMFT mixtures models among adolescents, there were single exposure effects for phosphorous and Vitamin K (Supplementary Figure S5). Specifically, individual changes in phosphorous and Vitamin K from their 25th to 75th percentiles were associated with 0.82 (95% CrI: 0.70, 0.95) and 0.87 (95% CrI: 0.78, 0.96) lower odds of DMFT respectively, when holding other nutrients at their median (Supplementary Table S6). When adjusting for sugar in the BKMR model, joint associations between nutrients and DFT or DMFT were consistent but attenuated (Figure 3). For example, a higher nutrient mixture was similarly associated with lower odds of DMFT in children 6–11 years old, but associations were no longer observed between the nutrient mixture and odds of DMFT in adolescents 12–19 years old.

## 4 Discussion

To our knowledge, this is the first contemporary study to examine associations between dietary intake of fat-soluble vitamins, vitamin C, calcium, phosphorus and phytates in relation to dental caries. Higher dietary intake of these nutrients was associated with less dental caries in youth, with phosphorus and certain fat-soluble vitamins being particularly important exposures. Specifically, higher dietary intake of vitamins A, E and K was associated with fewer dental caries in young children, older children and adolescents, respectively. Moreover, phosphorus and vitamin K appeared to be particularly important for dental health among children aged 6–11 years and adolescents when considered within the context of the other fat-soluble vitamins and minerals examined. Animal source foods including dairy products, muscle meat, organ meats and seafoods, can provide rich sources of phosphorus, as well as vitamins A and K2. Plant source foods such as seeds, beans and lentils are also high in phosphorus; orange fruits and vegetables and leafy green vegetables can be high in vitamin A, and green leafy vegetables and fermented foods can be high in vitamins K1 and K2, respectively (44–47). Additionally, avocados, as well as certain tree nuts and their oils are rich sources of vitamin E (48, 49). However, seeds, beans, nuts, legumes and whole grains may also contain antinutrients, such as phytates, that can interfere with nutrient absorption (44, 50, 51).

Findings of this study are consistent with historic studies demonstrating that fat-soluble vitamins contribute to healthy tooth formation and may even reverse dental caries. For example, Mellanby (52, 69) conducted experimental studies (52, 69) among puppies and rabbits in which she varied their diets with animal source foods rich in vitamins A, D and K2. She observed that puppies fed cod liver oil, butter, and suet (rich in vitamins A, D, and K2) had healthy tooth development, while those fed linseed oil, devoid of fat-soluble vitamins, had poor tooth development. Similarly, she observed that rabbits fed crushed oats, bran, lemon juice and calcium carbonate along with cod liver oil or egg yolks (rich in vitamins A and D) had normal tooth development and “healthy tooth calcification”. Conversely, rabbits fed the same diet but no cod liver oil or egg yolks had poor tooth calcification (52, 69). Consistently, in experimental studies of 7-year old children, Mellanby (54) and Mellanby & Pattinson (53, 55) found that children provided with a diet abundant in vitamins A and D for 6 months showed less initiation and spread of dental caries and experienced more hardening (improvement) of existing caries, when compared to children provided with less of these fat-soluble vitamins (53–55).

While the current study observed fewer dental caries among children aged 1–5 years who consumed more vitamin A in their diets, and adolescents who consumed more vitamin K in their diets, we did not observe an association of vitamin D intake with number of dental caries in any age group. Notably, participants in this study all had vitamin D intakes that were on average below the daily recommended intake ranges (42). Therefore, their vitamin D intakes may not have been sufficient or abundant enough to contribute to any potential dental health benefits. Specifically, the low average intake of vitamin D across all groups may have limited power to detect an association. Vitamin E intake also tended to be below the recommended level across all ages in this study; however, vitamin E intake may be underestimated as the amounts and types of fat added during cooking are often unknown and unaccounted for (56).

The potential importance of vitamin K for dental health observed in this study is mechanistically plausible and consistent with findings from historic studies. Vitamin K2 has been proposed as a nutrient that might help to prevent dental caries by increasing antioxidant defenses in the hypothalamus to blood sugar spikes that can contribute to dental caries (57). Furthermore, vitamin K2 acts as a co-factor of enzyme “gamma-glutamyl carboxylase” which can improve gamma-carboxylation of osteocalcin, a protein found in bones and teeth (58). Interestingly, historic observational and experimental human research conducted by Dr. Weston A. Price (59), the founder of the National Dental Association, reported a potential synergy between vitamin K2 and other fat-soluble vitamins in improving dental health. Dr. Price noted that when providing research participants with high vitamin cod liver oil (rich in vitamins A and D) along with high vitamin butter oil (rich in vitamin K2) these nutrients interacted to heal dental caries. Moreover, he highlighted the importance of calcium and phosphorus, as well as traditional dietary practices for dental caries prevention. He observed that switching from traditional diets rich in calcium, phosphorus and fat-soluble vitamins to modern diets was associated with an increase in dental caries; however, his research was not peer-reviewed [see (20) for a review of findings from peer-reviewed historic studies on nutrition and dental caries]. Nevertheless, two recent scientific reviews highlight the impact of contemporary dietary patterns and a switch toward processed foods deficient in essential nutrients, on the development of dental caries (17, 18). Similarly, another contemporary study conducted in Denmark found that a high intake of dairy foods, including milk (rich in calcium and phosphorus), may contribute to prevention of dental caries development during childhood and adolescence (14). While calcium was not associated with dental caries in this study, older children and adolescents tended to have intakes below the recommended level which could have limited any dental health benefits.

Contrary to our hypothesis, we observed that higher dietary intake of vitamin C was associated with *more* dental caries in young children. Fruit drinks contribute significantly to vitamin C intake among the US population, and these tend to be high in sugar (60, 61). Therefore, this association may be due to the high sugar content in fruit drinks consumed by young children, rather than vitamin C itself. Indeed, when we adjusted for sugar intake in our models, the positive association of vitamin C with dental caries was no longer statistically significant. Furthermore, research conducted in NHANES found that children aged 2–5 years who consumed fewer than five servings of fruits and vegetables (which tend to be high in vitamin C) (56, 62) per day had an increased likelihood of developing dental caries in their primary teeth (13). Additionally, vitamin C has well-documented oral health benefits (63). Unexpectedly, we also found that higher dietary phytate intake was associated with *fewer* dental caries in young children and not associated with dental caries among older children or adolescents. This may suggest that phytates may not be detrimental to dental health, or that some plant-based foods higher in phytates may also contribute to more favorable overall health effects that benefit dental health (64). Indeed, the capacity of phytates to reduce the solubility of fluoride, as well as calcium and phosphate, the main constituents of enamel, have been suggested as a mechanism by which phytates may prevent cavity formation (65). Furthermore, although some historic studies found phytates to have negative effects on tooth mineralization, many historic studies observed positive effects on caries from dietary protocols that included phytates within the context of a nutrient dense diet (20). Lastly, phytates are a main storage form of phosphorus (64), and therefore, the positive association between phytates and dental caries observed may have indirectly captured the potential benefit of phosphorus for dental health among younger children. However, in statistical analyses the observed relationship between phytates and DFT was not due to collinearity with phosphorus.

This study has several strengths. It utilized data from a large, nationally representative sample of the US population (NHANES 2011–2018) with standardized measures for dental caries assessment and daily dietary nutrient intake across two days. Additionally, we employed diverse and robust statistical approaches. Specifically, we employed negative binomial regression to account for overdispersion in the dental caries count data and applied the probit extension of BKMR to examine the joint associations of multiple nutrients with the probability of dental caries (40, 41). Combining these advanced statistical techniques allowed for a comprehensive and nuanced understanding of how dietary nutrients may impact dental health across different age groups. Further, all negative binomial regression analyses incorporated sampling weights, provided by NHANES, to reduce sampling bias. However, BKMR models did not integrate sampling weights or the survey design since the package has not been adapted yet.

However, this study has some limitations. First, dental caries was recorded using the DMFT index through a visual inspection technique (32) that does not use radiographs. This lack of radiographs could have underestimated the prevalence of dental caries, particularly interproximal lesions. Specifically, it may be difficult to distinguish between teeth lost due to caries compared to periodontal disease (32). Nevertheless, the DMFT is a recommended index (33), and has been widely used in numerous population-based studies (36, 37, 66, 67). In addition, the DMFT index has been shown to exhibit high reliability, with intra- and inter-examiner correlation coefficients exceeding 0.9 (68). Second, reverse causality cannot be ruled out due to the cross-sectional study design; however, given that this study included youth with relatively few dental caries on average, it is unlikely that their dental caries would have impacted dietary preferences. Lastly, the use of a self-report dietary recall method for ascertaining nutrient intake can contribute to recall bias; however, dietary misclassification in this study is likely to be non-systematic and bias associations toward the null.

## 5 Conclusion

This cross-sectional study found that higher dietary intake of phosphorus and fat-soluble vitamins A, E, and K was associated with fewer dental caries in a nationally representative sample of US children and adolescents. Contemporary prospective and experimental studies are needed to further examine these associations. Future research should also consider whether dietary interventions may be useful for mitigating dental caries, a common and costly public health issue.

## Data Availability

Publicly available datasets were analyzed in this study. This data can be found here: Data from National Health and Nutrition Examination Surveys (NHANES) are publicly available. The raw data supporting the conclusions of this article will be made available by the authors on reasonable request.
