# Supplementary material for: Higher fat-soluble vitamin and phosphorus intake are associated with less dental caries among children and adolescents in the United States, NHANES 2011–2018
Source: Front Oral Health. 2025 Jul 9;6:1617695. doi: 10.3389/froh.2025.1617695 (PMC12283692; doi:10.3389/froh.2025.1617695)
Supplement: Supplementary file 1 [file Table1.docx]

Supplementary Material

1. eMethods
2. **Supplementary Table S1:** Explanation of Dental codes utilized to derived DMFT and DFT scores
3. **Supplementary Table S2:** Adjusted negative binomial regression models; associations between quartiles of nutrients and dental caries across different age groups; Sensitivity analysis adjusting for BMI
4. **Supplementary Table S3:** Adjusted negative binomial regression models; associations between quartiles of nutrients and dental caries across different age groups; Sensitivity analysis adjusting for brushing frequency
5. **Supplementary Table S4:** Adjusted negative binomial regression models; associations between quartiles of nutrients and dental caries across different age groups; Sensitivity analysis adjusting for total sugar
6. **Supplementary Table S5:** Adjusted logistic regression models; associations between quartiles of nutrients and presence of dental caries across different age groups
7. **Supplementary Table S6**: BKMR results for the covariate adjusted single exposure effect estimates for each nutrient mixture on odds of DFT and DMFT
8. **Figure S1**: Univariate association of each nutrient on the probability of DFT (n=2,676) in children 1-5 years old or DMFT in children 6-11 (n= 3,214) and 12-19 years old (n=3,701), when compared to participants with no DFT or no DMFT.
9. **Figure S2:** Bivariate concentration-response curves evaluating associations between each nutrient on the probability of DFT relative to no DFT in children 1-5 years old (n=2,676).
10. **Figure S3:** Bivariate concentration-response curves evaluating associations between each nutrient on the probability of DMFT relative to no DMFT in children 6-11 years old (n= 3,214).
11. **Figure S4:** Bivariate concentration-response curves evaluating associations between each nutrient on the probability of DMFT relative to no DMFT in adolescent 12-19 years old (n=3,701).
12. **Figure S5:** Single-exposure health effects plots examining the change in a single nutrient on DFT or DMFT, relative to participants with no DFT or DMFT.

**eMethods**

**NHANES Survey Procedures:** NHANES utilizes a sophisticated, multistage probability sampling technique, to gather de-identified data on behavioral, demographic, and environmental factors. Participants were initially interviewed at their residences regarding their medical history, health behaviors, and associated risk factors. Subsequently, they were referred to a mobile examination center (MEC) for clinical assessments, including a dental exam, nutritional evaluations, and the collection of biological specimens (NHANES, 2020).

**Negative Binomial Regression Model Fit:** We compared model fit of negative binomial regression to Poisson regression and zero- inflated Poisson regression. Negative binomial regression provided the lowest Akaike's information criteria (AIC) and Bayesian information criteria (BIC) scores when compared to the other count modelling approaches, indicating better model fit.

**BKMR Data Preparation:** Prior to BKMR modeling, all nutrients were offset by 0.1, natural log-transformed, mean-centered, and standard deviation scaled. All continuous covariates were also mean-centered and scaled to one standard deviation. The Markov chain Monte Carlo sampler was set to obtain 20,000 iterations. The first half were used as burn-in and chains were thinned to every 10^th^ iteration. Convergence was visually assessed and confirmed using trace plots.

**BKMR univariate nutrient associations and pairwise interactions.** In supplemental analyses, we examined the univariate associations between each nutrient on odds of DFT or DMFT, while holding all other nutrients at their median. We further assessed these associations by examining a change in nutrients from their 25th to 75th percentile, while holding other nutrients at their 25th, 50th, and 75th percentile. We also examined pairwise interactions between nutrients when varying a second nutrient to its 25th, 50th, and 75th percentile, holding other nutrients to their median.

| Supplementary Table S1: Explanation of Dental codes utilized to derived DMFT and DFT scores | |
| --- | --- |
| NHANES Oral Health  Dental codes | Explanation |
| Z | Permanent tooth with surface condition/dental carious surface condition |
| F | Permanent tooth with a restored surface condition ^a^ |
| K | Primary tooth with surface condition/dental carious surface condition |
| A | Primary tooth with a restored surface condition ^a^ |
| E | Missing due to dental disease (caries/periodontal disease) |
| M | Missing due to other causes (orthodontic/traumatic or other non-disease) |
| R | Missing due to dental disease but replaced by a fixed restoration |
| X | Missing due to other causes but replaced by a fixed restoration |
| P | Missing due to dental disease but replaced by a removable restoration |
| Q | Missing due to other causes but replaced by a removable restoration |
| J | Permanent root tip is present, but no restorative replacement is present |
| T | Permanent root tip is present, but a restorative replacement is present |
| ^a^ “F” and “A” codes are introduced as an additional codes in 2015 and onward data sets representing permanent and primary tooth restoration respectively. Prior 2015, codes “Z” and “K” corresponded to permanent and primary tooth restoration or caries respectively. | |

| Supplementary Table S2: Adjusted negative binomial regression models; associations between quartiles of nutrients and dental caries across different age groups; Sensitivity analysis adjusting for BMI | | | | | | |
| --- | --- | --- | --- | --- | --- | --- |
| Dietary Nutrients Quartiles | DFT score for primary teeth  (No. of teeth with decay or filling) | | DMFT score for primary and permanent teeth  (No. of teeth with decay, missing or filling) | | | |
|  | **Children Age 1-5 years**  **(n=1,610)** | | **Children Age 6-11 years**  **(n=2,584)** | | **Adolescents Aged 12-19 years**  **(n=2,865)** | |
|  | Rate Ratios  RR (95% CI) | p-value | Rate Ratios  RR (95% CI) | p-value | Rate Ratios  RR (95% CI) | p-value |
| Calcium |  |  |  |  |  |  |
| Q1 (31.5-594) | Reference |  | Reference |  | Reference |  |
| Q2 (595.5-842) | 0.74 (0.44-1.24) | 0.249 | 0.84 (0.69-1.16) | 0.240 | 0.89 (0.80-1.17) | 0.327 |
| Q3 (842.5-1148.5) | 0.84 (0.50-1.42) | 0.522 | 0.79 (0.60-1.09) | 0.111 | 1.03 (0.77-1.13) | 0.823 |
| Q4(1149-5237.5) | 0.79 (0.42-1.50) | 0.481 | 0. 86 (0.65-1.16) | 0.395 | 0.86 (0.68-1.06) | 0.238 |
| Phosphorus |  |  |  |  |  |  |
| Q1 (63-888) | Reference |  | Reference |  | Reference |  |
| Q2(888.5-1180.5) | 0.48 (0.31-0.76) | **0.001** | 0.81 (0.61-1.04) | 0.110 | 1.04 (0.84-1.2) | 0.715 |
| Q3(1181-1531.5) | 0.42 (0.29-0.94) | **0.012** | 0.84 (0.64-1.08) | 0.236 | 0.95 (0.73-1.23) | 0.697 |
| Q4(1532.5-5535.5) | 0.51 (0.25-1.20) | 0.109 | 0.65 (0.40-0.84) | **0.038** | 0.89 (0.53-1.26) | 0.518 |
| Vitamin A |  |  |  |  |  |  |
| Q1 (1-327.5) | Reference |  | Reference |  | Reference |  |
| Q2 (328-511.5) | 0.73 (0.42-1.09) | 0.111 | 0.92 (0.74-1.14) | 0.677 | 0.97 (0.77-1.10) | 0.803 |
| Q3 (512-754.5) | 0.64 (0.37-1.12) | 0.118 | 1.01 (0.80-1.28) | 0.916 | 0.86 (0.73-1.07) | 0.197 |
| Q4 (755-7773.5) | 0.72 (0.34-1.21) | 0.166 | 1.06 (0.72-1.25) | 0.698 | 0.96 (0.74-1.11) | 0.797 |
| Vitamin C |  |  |  |  |  |  |
| Q1 (0-31.4) | Reference |  | Reference |  | Reference |  |
| Q2 (31.45-63.15) | 1.53 (0.78-3.03) | 0.214 | 0.75 (0.57-0.98) | **0.033** | 0.92 (0.81-1.17) | 0.382 |
| Q3 (63.2-108.05) | 1.46 (0.74-2.87) | 0.270 | 1.00 (0.81-1.31) | 0.947 | 0.81 (0.73-1.07) | 0.059 |
| Q4 (108-1635.85) | 2.76 (1.31-5.80) | **0.007** | 0.83 (0.66-1.05) | 0.134 | 0.81 (0.72-1.07) | 0.065 |
| Vitamin D |  |  |  |  |  |  |
| Q1 (0-2.1) | Reference |  | Reference |  | Reference |  |
| Q2 (2.15-4) | 1.98 (1.00-3.92) | 0.049 | 1.16 (0.89-1.51) | 0.347 | 1.44 (1.18-1.48) | **<0.001** |
| Q3 (4.05-6.7) | 1.08 (0.48-1.43) | 0.498 | 1.03 (0.74-1.26) | 0.836 | 1.16 (0.95-1.24) | 0.126 |
| Q4 (6.75-47.3) | 1.29 (0.42-1.40) | 0.380 | 1.12 (0.77-1.37) | 0.469 | 1.18 (0.94-1.27) | 0.142 |
| Vitamin E |  |  |  |  |  |  |
| Q1 (0.4-4.71) | Reference |  | Reference |  | Reference |  |
| Q2 (4.72-6.74) | 1.19 (0.72-1.57) | 0.443 | 0.88 (0.64-1.12) | 0.312 | 1.10 (0.87-1.29) | 0.587 |
| Q3 (6.75-9.59) | 1.15 (0.67-1.73) | 0.553 | 0.86 (0.62-1.12) | 0.259 | 1.08 (0.79-1.20) | 0.814 |
| Q4 (9.6-115.03) | 1.52 (0.70-2.46) | 0.210 | 0.73 (0.54-1.02) | 0.066 | 1.10 (0.81-1.29) | 0.861 |
| Vitamin K |  |  |  |  |  |  |
| Q1 (0.95-39.65) | Reference |  | Reference |  | Reference |  |
| Q2 (39.7-64.65) | 0.86 (0.59-1.24) | 0.427 | 0.85 (0.69-1.04) | 0.123 | 0.86 (0.68-0.97) | 0.134 |
| Q3 (64.7-111.2) | 1.80 (0.95-3.43) | 0.070 | 0.83 (0.66-1.04) | 0.107 | 0.84 (0.67-1.00) | 0.164 |
| Q4 (111.25-1828.65) | 1.29 (0.69-2.39) | 0.426 | 0.78 (0.68-1.15) | 0.366 | 0.84 (0.69-1.05) | 0.162 |
| Phytate |  |  |  |  |  |  |
| Q1 (0-342.43) | Reference |  | Reference |  | Reference |  |
| Q2 (342.53-566.41) | 0.75 (0.44-1.06) | 0.270 | 1.02 (0.82-1.25) | 0.665 | 0.98 (0.80-1.20) | 0.556 |
| Q3 (566.62-908.66) | 0.44 (0.31-0.72) | **<0.001** | 0.87 (0.70-1.08) | 0.421 | 0.95 (0.78-1.17) | 0.978 |
| Q4 (908.71-7641.07) | 0.32 (0.21-0.65) | **<0.001** | 0.84 (0.66-1.06) | 0.164 | 0.85 (0.63-1.07) | 0.673 |
| The estimates, 95% CIs and p-values were calculated using the 2 days dietary sampling weights to account for the complex survey design of NHANES  For models with phytates the day 1 dietary weights were used  All models are adjusted for age, sex, race/ethnicity, ratio of family income to poverty, total energy intake, dental visits and BMI  Bold values represent significant p-value <0.05 | | | | | | |

| Supplementary Table S3: Adjusted negative binomial regression models; associations between quartiles of nutrients and dental caries across different age groups; Sensitivity analysis adjusting for brushing frequency | | | | | | |
| --- | --- | --- | --- | --- | --- | --- |
| Dietary Nutrients Quartiles | DFT score for primary teeth  (No. of teeth with decay or filling) | | DMFT score for primary and permanent teeth  (No. of teeth with decay, missing or filling) | | | |
|  | **Children Age 1-5 years**  **(n=955)** | | **Children Age 6-11 years**  **(n=2,191)** | | **Adolescents Aged 12-19 years (n=2,670)** | |
|  | Rate Ratios  RR (95% CI) | p-value | Rate Ratios  RR (95% CI) | p-value | Rate Ratios  RR (95% CI) | p-value |
| Calcium |  |  |  |  |  |  |
| Q1 (31.5-594) | Reference |  | Reference |  | Reference |  |
| Q2 (595.5-842) | 0.90 (0.44-1.15) | 0.733 | 0.91 (0.69-1.16) | 0.566 | 1.04 (0.80-1.17) | 0.752 |
| Q3 (842.5-1148.5) | 0.74 (0.49-1.27) | 0.283 | 0.85 (0.66-1.09) | 0.268 | 0.88 (0.77-1.13) | 0.254 |
| Q4(1149-5237.5) | 0.72 (0.41-1.32) | 0.363 | 0. 87 (0.65-1.16) | 0.234 | 0.86 (0.68-1.06) | 0.217 |
| Phosphorus |  |  |  |  |  |  |
| Q1 (63-888) | Reference |  | Reference |  | Reference |  |
| Q2(888.5-1180.5) | 0.46 (0.35-0.76) | **0.002** | 0.72 (0.61-0.97) | **0.026** | 0.81 (0.73-1.09) | 0.071 |
| Q3(1181-1531.5) | 0.45 (0.29-0.94) | **0.021** | 0.78 (0.64-1.08) | 0.098 | 0.68 (0.52-0.91) | **0.009** |
| Q4(1532.5-5535.5) | 0.54 (0.25-1.20) | 0.230 | 0.49 (0.40-0.84) | **0.001** | 0.61 (0.43-0.88) | **0.009** |
| Vitamin A |  |  |  |  |  |  |
| Q1 (1-327.5) | Reference |  | Reference |  | Reference |  |
| Q2 (328-511.5) | 0.83 (0.42-1.09) | 0.496 | 0.89 (0.74-1.14) | 0.427 | 0.88 (0.77-1.10) | 0.239 |
| Q3 (512-754.5) | 0.64 (0.37-1.11) | 0.115 | 0.97 (0.80-1.28) | 0.828 | 0.94 (0.73-1.07) | 0.609 |
| Q4 (755-7773.5) | 0.51 (0.27-0.93) | **0.028** | 0.93 (0.72-1.25) | 0.662 | 0.87 (0.74-1.11) | 0.226 |
| Vitamin C |  |  |  |  |  |  |
| Q1 (0-31.4) | Reference |  | Reference |  | Reference |  |
| Q2 (31.45-63.15) | 1.17 (0.72-2.11) | 0.652 | 0.84 (0.66-1.07) | 0.308 | 1.04 (0.81-1.17) | 0.721 |
| Q3 (63.2-108.05) | 1.17 (0.72-2.16) | 0.639 | 1.03 (0.81-1.31) | 0.547 | 0.95 (0.73-1.07) | 0.636 |
| Q4 (108-1635.85) | 0.99 (0.51-3.60) | 0.967 | 0.83 (0.66-1.05) | 0.204 | 0.94 (0.72-1.07) | 0.639 |
| Vitamin D |  |  |  |  |  |  |
| Q1 (0-2.1) | Reference |  | Reference |  | Reference |  |
| Q2 (2.15-4) | 0.62 (0.51-1.87) | 0.191 | 1.16 (0.89-1.51) | 0.431 | 1.14 (0.94-1.39) | 0.092 |
| Q3 (4.05-6.7) | 0.79 (0.48-1.43) | 0.462 | 0.96 (0.74-1.26) | 0.524 | 1.03 (0.86-1.24) | 0.820 |
| Q4 (6.75-47.3) | 0.72 (0.42-1.40) | 0.334 | 1.02 (0.77-1.37) | 0.645 | 1.04 (0.85-1.27) | 0.867 |
| Vitamin E |  |  |  |  |  |  |
| Q1 (0.4-4.71) | Reference |  | Reference |  | Reference |  |
| Q2 (4.72-6.74) | 0.85 (0.72-1.57) | 0.535 | 0.77 (0.58-0.99) | **0.050** | 1.06 (0.87-1.37) | 0.457 |
| Q3 (6.75-9.59) | 1.39 (0.67-1.73) | 0.260 | 0.82 (0.62-1.12) | 0.230 | 0.93 (0.74-1.19) | 0.594 |
| Q4 (9.6-115.03) | 1.82 (0.70-2.46) | 0.178 | 0.81 (0.54-1.20) | 0.295 | 1.06 (0.81-1.38) | 0.662 |
| Vitamin K |  |  |  |  |  |  |
| Q1 (0.95-39.65) | Reference |  | Reference |  | Reference |  |
| Q2 (39.7-64.65) | 0.99 (0.64-1.26) | 0.989 | 0.74 (0.58-0.95) | **0.017** | 0.85 (0.69-1.04) | 0.117 |
| Q3 (64.7-111.2) | 1.10 (0.93-2.94) | 0.747 | 0.82 (0.60-1.04) | 0.176 | 0.86 (0.68-1.08) | 0.195 |
| Q4 (111.25-1828.65) | 1.91 (0.80-2.86) | 0.141 | 0.96 (0.68-1.15) | 0.838 | 0.86 (0.67-1.05) | 0.245 |
| Phytate |  |  |  |  |  |  |
| Q1 (0-342.43) | Reference |  | Reference |  | Reference |  |
| Q2 (342.53-566.41) | 0.56 (0.44-0.91) | **0.020** | 1.09 (0.82-1.25) | 0.525 | 0.96 (0.76-1.20) | 0.729 |
| Q3 (566.62-908.66) | 0.40 (0.31-0.72) | **<0.001** | 1.11 (0.70-1.08) | 0.474 | 0.90 (0.70-1.17) | 0.430 |
| Q4 (908.71-7641.07) | 0.32 (0.21-0.65) | **<0.001** | 0.97 (0.66-1.06) | 0.835 | 0.78 (0.59-1.03) | 0.083 |
| The estimates, 95% CIs and p-values were calculated using the 2 days dietary sampling weights to account for the complex survey design of NHANES  For models with phytates the day 1 dietary weights were used  All models are adjusted for age, sex, race/ethnicity, ratio of family income to poverty, total energy intake, dental visits, and brushing frequency  Bold values represent significant p-value <0.05 | | | | | | |

| Supplementary Table S4: Adjusted negative binomial regression models; associations between quartiles of nutrients and dental caries across different age groups; Sensitivity analysis adjusting for total sugar | | | | | | |
| --- | --- | --- | --- | --- | --- | --- |
| Dietary Nutrients Quartiles | **DFT score for primary teeth**  (No. of teeth with decay or filling) | | **DMFT score for primary and permanent teeth**  (No. of teeth with decay, missing or filling) | | | |
|  | **Children Age 1-5 years**  **(n=2,676)** | | **Children Age 6-11 years**  **(n=3,214)** | | **Adolescents Aged 12-19 years (n=3,701)** | |
|  | Rate Ratios  RR (95% CI) | p-value | Rate Ratios  RR (95% CI) | p-value | Rate Ratios  RR (95% CI) | p-value |
| Calcium |  |  |  |  |  |  |
| Q1 (31.5-594) | Reference |  | Reference |  | Reference |  |
| Q2 (595.5-842) | 0.71 (0.44-1.15) | 0.162 | 0.89 (0.69-1.16) | 0.394 | 0.97 (0.80-1.17) | 0.740 |
| Q3 (842.5-1148.5) | 0.78 (0.48-1.26) | 0.308 | 0.84 (0.65-1.09) | 0.191 | 0.93 (0.77-1.13) | 0.461 |
| Q4(1149-5237.5) | 0.74 (0.41-1.33) | 0.315 | 0. 87 (0.65-1.16) | 0.343 | 0.85 (0.68-1.06) | 0.138 |
| Phosphorus |  |  |  |  |  |  |
| Q1 (63-888) | Reference |  | Reference |  | Reference |  |
| Q2(888.5-1180.5) | 0.52 (0.35-0.77) | **0.001** | 0.77 (0.61-0.97) | **0.032** | 0.90 (0.73-1.09) | 0.270 |
| Q3(1181-1531.5) | 0.54 (0.29-0.98) | **0.042** | 0.84 (0.64-1.08) | 0.221 | 0.80 (0.63-1.02) | 0.069 |
| Q4(1532.5-5535.5) | 0.60 (0.27-1.34) | 0.217 | 0.64 (0.40-0.84) | **0.007** | 0.75 (0.53-1.03) | 0.080 |
| Vitamin A |  |  |  |  |  |  |
| Q1 (1-327.5) | Reference |  | Reference |  | Reference |  |
| Q2 (328-511.5) | 0.66 (0.41-1.06) | 0.088 | 0.92 (0.74-1.14) | 0.488 | 0.91 (0.81-1.16) | 0.320 |
| Q3 (512-754.5) | 0.58 (0.36-0.93) | **0.024** | 1.00 (0.80-1.28) | 0.987 | 0.88 (0.71-1.03) | 0.191 |
| Q4 (755-7773.5) | 0.67 (0.35-1.27) | 0.216 | 0.94 (0.61-1.00) | 0.677 | 0.90 (0.68-1.01) | 0.309 |
| Vitamin C |  |  |  |  |  |  |
| Q1 (0-31.4) | Reference |  | Reference |  | Reference |  |
| Q2 (31.45-63.15) | 1.20 (0.72-2.11) | 0.500 | 0.82 (0.66-1.04) | 0.110 | 0.97 (0.81-1.17) | 0.748 |
| Q3 (63.2-108.05) | 1.21 (0.72-2.16) | 0.505 | 0.99 (0.78-1.26) | 0.968 | 0.85 (0.73-1.07) | 0.102 |
| Q4 (108-1635.85) | 1.80 (0.90-3.61) | 0.098 | 0.78 (0.61-1.00) | 0.055 | 0.83 (0.72-1.07) | 0.063 |
| Vitamin D |  |  |  |  |  |  |
| Q1 (0-2.1) | Reference |  | Reference |  | Reference |  |
| Q2 (2.15-4) | 0.98 (0.51-1.86) | 0.951 | 1.15 (0.89-1.51) | 0.299 | 1.24 (1.05-1.48) | **0.011** |
| Q3 (4.05-6.7) | 0.84 (0.48-1.46) | 0.538 | 0.94 (0.74-1.26) | 0.702 | 1.03 (0.86-1.24) | 0.790 |
| Q4 (6.75-47.3) | 0.77 (0.42-1.42) | 0.397 | 0.99 (0.77-1.37) | 0.976 | 1.04 (0.85-1.27) | 0.786 |
| Vitamin E |  |  |  |  |  |  |
| Q1 (0.4-4.71) | Reference |  | Reference |  | Reference |  |
| Q2 (4.72-6.74) | 1.13 (0.72-1.57) | 0.541 | 0.79 (0.64-0.98) | **0.045** | 1.06 (0.87-1.29) | 0.475 |
| Q3 (6.75-9.59) | 1.16 (0.67-1.73) | 0.553 | 0.79 (0.62-1.00) | 0.094 | 0.97 (0.79-1.20) | 0.983 |
| Q4 (9.6-115.03) | 1.54 (0.70-2.46) | 0.191 | 0.73 (0.54-0.97) | 0.058 | 1.02 (0.81-1.29) | 0.482 |
| Vitamin K |  |  |  |  |  |  |
| Q1 (0.95-39.65) | Reference |  |  | Reference |  | Reference |
| Q2 (39.7-64.65) | 0.94 (0.64-1.26) | 0.710 | 0.85 (0.69-1.04) | 0.137 | 0.82 (0.68-0.97) | **0.038** |
| Q3 (64.7-111.2) | 1.76 (0.93-2.94) | 0.058 | 0.83 (0.66-1.04) | 0.178 | 0.82 (0.67-1.00) | 0.110 |
| Q4 (111.25-1828.65) | 1.60 (0.80-2.86) | 0.166 | 0.89 (0.68-1.15) | 0.487 | 0.85 (0.69-1.05) | 0.274 |
| Phytate |  |  |  |  |  |  |
| Q1 (0-342.43) | Reference |  | Reference |  | Reference |  |
| Q2 (342.53-566.41) | 0.69 (0.44-1.06) | 0.102 | 1.05 (0.84-1.30) | 0.643 | 0.99 (0.81-1.21) | 0.948 |
| Q3 (566.62-908.66) | 0.49 (0.31-0.72) | **0.001** | 0.91 (0.73-1.14) | 0.416 | 0.98 (0.80-1.20) | 0.881 |
| Q4 (908.71-7641.07) | 0.39 (0.21-0.65) | **0.001** | 0.90 (0.70-1.15) | 0.410 | 0.89 (0.71-1.11) | 0.297 |
| The estimates, 95% CIs and p-values were calculated using the 2 days dietary sampling weights to account for the complex survey design of NHANES  For models with phytates the day 1 dietary weights were used  All models are adjusted for age, sex, race/ethnicity, ratio of family income to poverty, total energy intake, dental visits, and total sugar intake  Bold values represent significant p-value <0.05 | | | | | | |

| Supplementary Table S5: Adjusted logistic regression models; associations between quartiles of nutrients and presence of dental caries across different age groups | | | | | | |
| --- | --- | --- | --- | --- | --- | --- |
| Dietary Nutrients Quartiles | DFT score for primary teeth  DFT ≥1 | | DMFT score for permanent teeth  DMFT ≥1 | | | |
|  | **Children Age 1-5 years**  (n=2,676) | | **Children Age 6-11 years**  (n=3,214) | | **Adolescents Aged 12-19 years**  (n=3,701) | |
|  | Odds Ratios  OR (95% CI) | p-value | Odds Ratios  OR (95% CI) | p-value | Odds Ratios  OR (95% CI) | p-value |
| Calcium |  |  |  |  |  |  |
| Q1 (31.5-594) | Reference |  | Reference |  | Reference |  |
| Q2 (595.5-842) | 0.95 (0.59-1.53) | 0.829 | 0.98 (0.68-1.41) | 0.909 | 0.83 (0.60-1.13) | 0.233 |
| Q3 (842.5-1148.5) | 0.85 (0.53-1.35) | 0.485 | 0.83 (0.58-1.19) | 0.306 | 0.83 (0.59-1.16) | 0.275 |
| Q4(1149-5237.5) | 0.79 (0.44-1.39) | 0.407 | 0.70 (0.47-1.05) | 0.089 | 0.66 (0.45-0.95) | **0.026** |
| Phosphorus |  |  |  |  |  |  |
| Q1 (63-888) | Reference |  | Reference |  | Reference |  |
| Q2(888.5-1180.5) | 0.53 (0.33-0.84) | **0.008** | 0.71 (0.50-1.00) | **0.052** | 0.83 (0.60-1.15) | 0.265 |
| Q3(1181-1531.5) | 0.41 (0.21-0.80) | **0.009** | 0.67 (0.45-0.99) | **0.043** | 0.68 (0.47-0.99) | **0.043** |
| Q4(1532.5-5535.5) | 0.46 (0.17-1.27) | 0.132 | 0.45 (0.27-0.76) | **0.003** | 0.51 (0.32-0.83) | **0.006** |
| Vitamin A |  |  |  |  |  |  |
| Q1 (1-327.5) | Reference |  | Reference |  | Reference |  |
| Q2 (328-511.5) | 0.94 (0.59-1.51) | 0.810 | 0.83 (0.59-1.18) | 0.296 | 0.83 (0.61-1.12) | 0.225 |
| Q3 (512-754.5) | 0.72 (0.45-1.17) | 0.186 | 0.78 (0.55-1.13) | 0.190 | 0.93 (0.67-1.28) | 0.641 |
| Q4 (755-7773.5) | 0.77 (0.44-1.34) | 0.359 | 0.72 (0.49-1.06) | 0.097 | 0.81 (0.58-1.14) | 0.228 |
| Vitamin C |  |  |  |  |  |  |
| Q1 (0-31.4) | Reference |  | Reference |  | Reference |  |
| Q2 (31.45-63.15) | 1.10 (0.64-1.89) | 0.740 | 0.85 (0.61-1.18) | 0.325 | 0.88 (0.65-1.61) | 0.357 |
| Q3 (63.2-108.05) | 1.26 (0.73-2.16) | 0.402 | 1.06 (0.75-1.49) | 0.733 | 0.86 (0.63-1.16) | 0.317 |
| Q4 (108-1635.85) | 2.31 (1.29-4.14) | **0.005** | 1.03 (0.72-1.47) | 0.864 | 0.75 (0.54-1.03) | 0.075 |
| Vitamin D |  |  |  |  |  |  |
| Q1 (0-2.1) | Reference |  | Reference |  | Reference |  |
| Q2 (2.15-4) | 0.94 (0.47-1.88) | 0.852 | 1.27 (0.85-1.89) | 0.239 | 1.01 (0.75-1.36) | 0.949 |
| Q3 (4.05-6.7) | 0.93 (0.47-1.82) | 0.827 | 1.05 (0.72-1.54) | 0.789 | 0.99 (0.73-1.35) | 0.973 |
| Q4 (6.75-47.3) | 0.91 (0.44-1.91) | 0.810 | 0.91 (0.61-1.36) | 0.639 | 0.90 (0.65-1.26) | 0.551 |
| Vitamin E |  |  |  |  |  |  |
| Q1 (0.4-4.71) | Reference |  | Reference |  | Reference |  |
| Q2 (4.72-6.74) | 1.08 (0.69-1.69) | 0.734 | 0.65 (0.48-0.90) | **0.008** | 1.22 (0.89-1.67) | 0.220 |
| Q3 (6.75-9.59) | 1.36 (0.76-2.43) | 0.300 | 0.63 (0.44-0.91) | **0.013** | 1.12 (0.80-1.58) | 0.511 |
| Q4 (9.6-115.03) | 2.04 (1.02-4.06) | **0.043** | 0.68 (0.44-1.06) | 0.091 | 1.12 (0.75-1.67) | 0.577 |
| Vitamin K |  |  |  |  |  |  |
| Q1 (0.95-39.65) | Reference |  | Reference |  | Reference |  |
| Q2 (39.7-64.65) | 1.05 (0.72-1.55) | 0.789 | 0.83 (0.62-1.11) | 0.208 | 0.73 (0.55-0.96) | **0.027** |
| Q3 (64.7-111.2) | 1.66 (1.01-2.72) | **0.045** | 0.74 (0.53-1.02) | 0.063 | 0.67 (0.50-0.97) | **0.031** |
| Q4 (111.25-1828.65) | 1.81 (0.94-3.51) | 0.078 | 1.02 (0.68-1.53) | 0.927 | 0.81 (0.56-1.16) | 0.243 |
| Phytate |  |  |  |  |  |  |
| Q1 (0-342.43) | Reference |  | Reference |  | Reference |  |
| Q2 (342.53-566.41) | 0.74 (0.44-1.06) | 0.149 | 1.09 (0.81-1.48) | 0.562 | 0.95 (0.71-1.28) | 0.757 |
| Q3 (566.62-908.66) | 0.64 (0.31-0.72) | **0.048** | 0.80 (0.58-1.09) | 0.163 | 1.03 (0.76-1.41) | 0.835 |
| Q4 (908.71-7641.07) | 0.38 (0.21-0.65) | **0.001** | 0.81 (0.57-1.15) | 0.232 | 0.97 (0.34-0.84) | 0.893 |
| The estimates, 95% CIs and p-values were calculated using the 2 days dietary sampling weights to account for the complex survey design of NHANES  For models with phytates the day 1 dietary weights were used  All models are adjusted for age, sex, race/ethnicity, ratio of family income to poverty, total energy intake, and dental visits  Bold values represent significant p-value <0.05 | | | | | | |

| **Supplementary Table S6**: **BKMR results for the covariate adjusted single exposure effect estimates for each nutrient mixture on odds of DFT and DMFT** | | | | |
| --- | --- | --- | --- | --- |
|  |  | Children 1-5 years old  OR (95% CrI) | Children 6-11 years old  OR (95% CrI) | Adolescent 12-19 years old  OR (95% CrI) |
| Individual Nutrient Effect Estimates^a^ | |  |  |  |
|  | Calcium | 1.02 (0.87, 1.19) | 0.98 (0.83, 1.15) | 1.11 (0.96, 1.28) |
|  | Phosphorous | 0.75 (0.63, 0.89) | 0.79 (0.67, 0.94) | 0.82 (0.70, 0.95) |
|  | Vitamin A | 0.95 (0.85, 1.07) | 0.95 (0.85, 1.06) | 0.98 (0.87, 1.09) |
|  | Vitamin C | 1.25 (1.13, 1.38) | 1.05 (0.97, 1.15) | 1.04 (0.95, 1.14) |
|  | Vitamin D | 1.01 (0.90, 1.13) | 1.04 (0.94, 1.15) | 1.03 (0.93, 1.14) |
|  | Vitamin E | 1.03 (0.93, 1.15) | 0.92 (0.83, 1.02) | 0.98 (0.89, 1.09) |
|  | Vitamin K | 1.01 (0.91, 1.11) | 0.98 (0.89, 1.08) | 0.87 (0.78, 0.96) |
|  | Phytate | 0.94 (0.85, 1.04) | 1.02 (0.94, 1.11) | 1.02 (0.94, 1.09) |
| BKMR, Bayesian Kernal Machine Regression  ^a^Odds ratio for DFT or DMFT when concentrations of an individual nutrient are held at the 75^th^ percentile compared to the 25^th^ percentile, while holding all other nutrients at the median. Credible intervals (CrI)  Models were adjusted for age, sex, race and ethnicity, ratio of family income to poverty, total energy, and last dental visit. | | | | |

|  |
| --- |
| **Figure S1:** Univariate association of each nutrient on the probability of DFT (n=2,676) in children 1-5 years old or DMFT in children 6-11 (n= 3,214) and 12-19 years old (n=3,701), when compared to participants with no DFT or no DMFT. Figures illustrate the probit model estimates and 95% credible intervals of exposure-response associations between each nutrient on the probability of DFT or DMFT vs. no DFT or DMFT, while setting all other metals to their median. Models were adjusted for age, sex, race and ethnicity, ratio of family income to poverty, total energy, and last dental visit. |

| 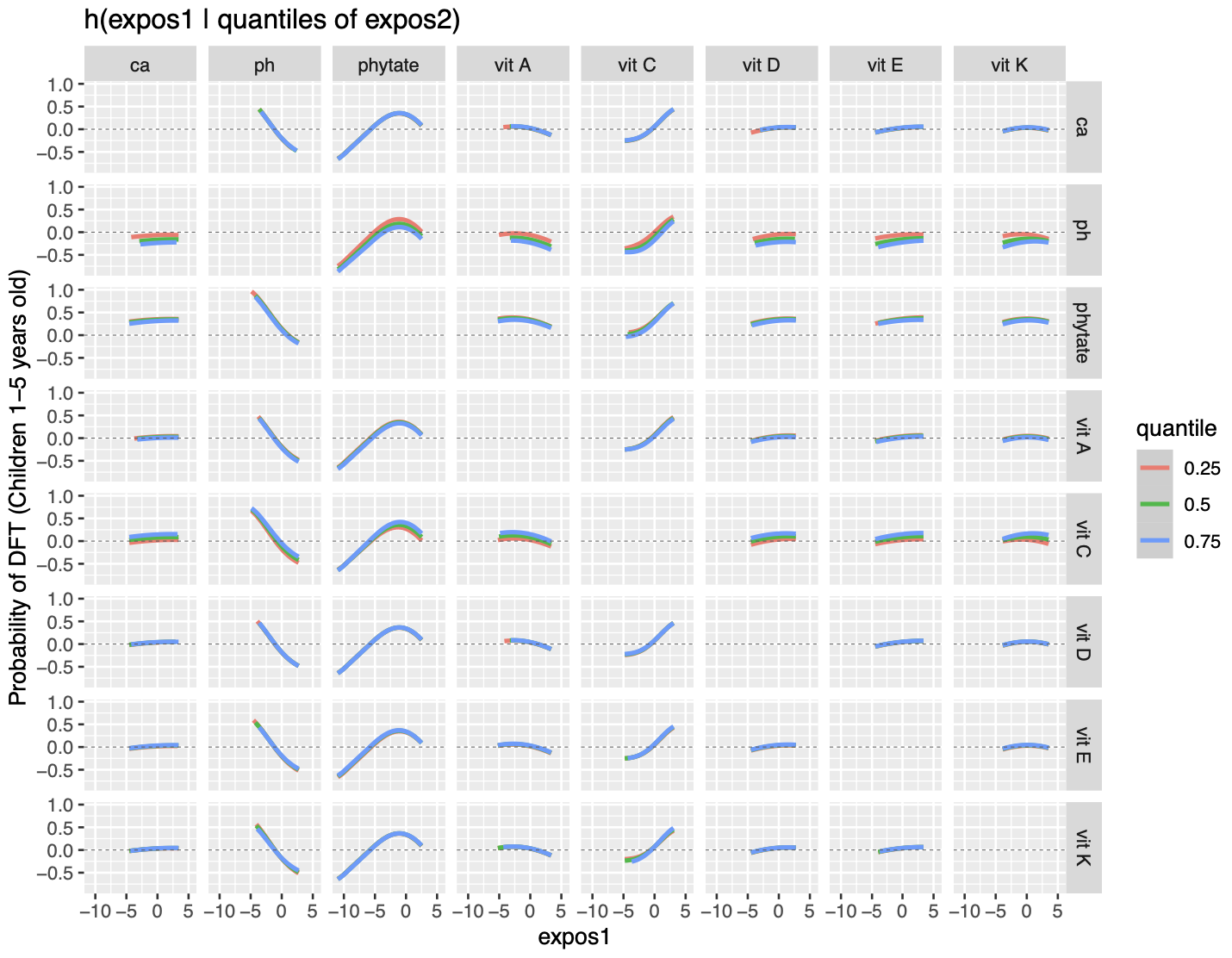 |
| --- |
| **Figure S2:** Bivariate concentration-response curves evaluating associations between each nutrient on the probability of DFT relative to no DFT in children 1-5 years old (n=2,676). Figures illustrate the probit model estimates and 95% credible intervals for bivariate associations between all nutrients on probability of DFT, while setting a second nutrient to its 25^th^, 50^th^, and 75^th^ percentile and all other nutrients to their median. Models were adjusted for age, sex, race and ethnicity, ratio of family income to poverty, total energy, and last dental visit. |

| 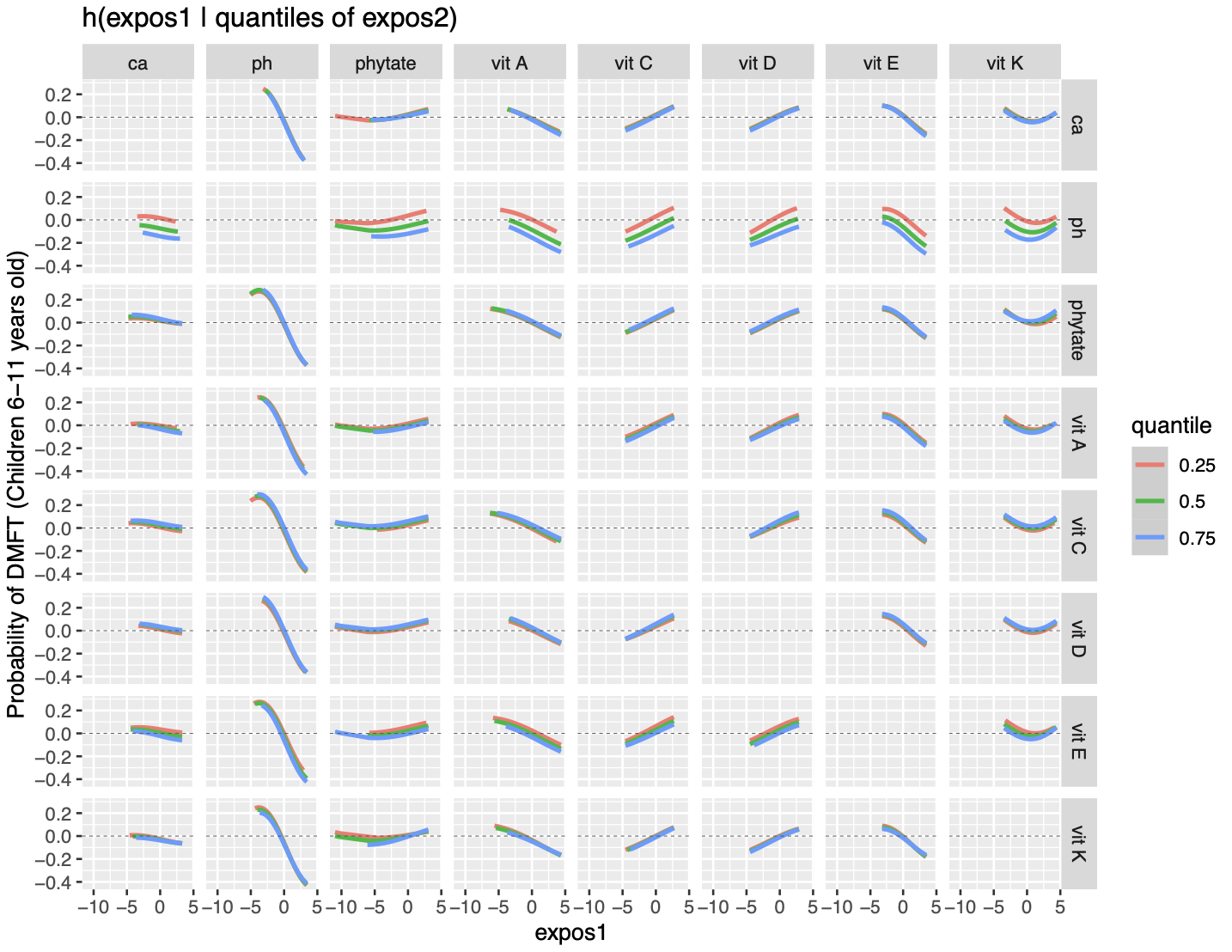 |
| --- |
| **Figure S3:** Bivariate concentration-response curves evaluating associations between each nutrient on the probability of DMFT relative to no DMFT in children 6-11 years old (n= 3,214). Figures illustrate the probit model estimates and 95% credible intervals for bivariate associations between all nutrients on probability of DMFT, while setting a second nutrient to its 25^th^, 50^th^, and 75^th^ percentile and all other nutrients to their median. Models were adjusted for age, sex, race and ethnicity, ratio of family income to poverty, total energy, and last dental visit. |

| 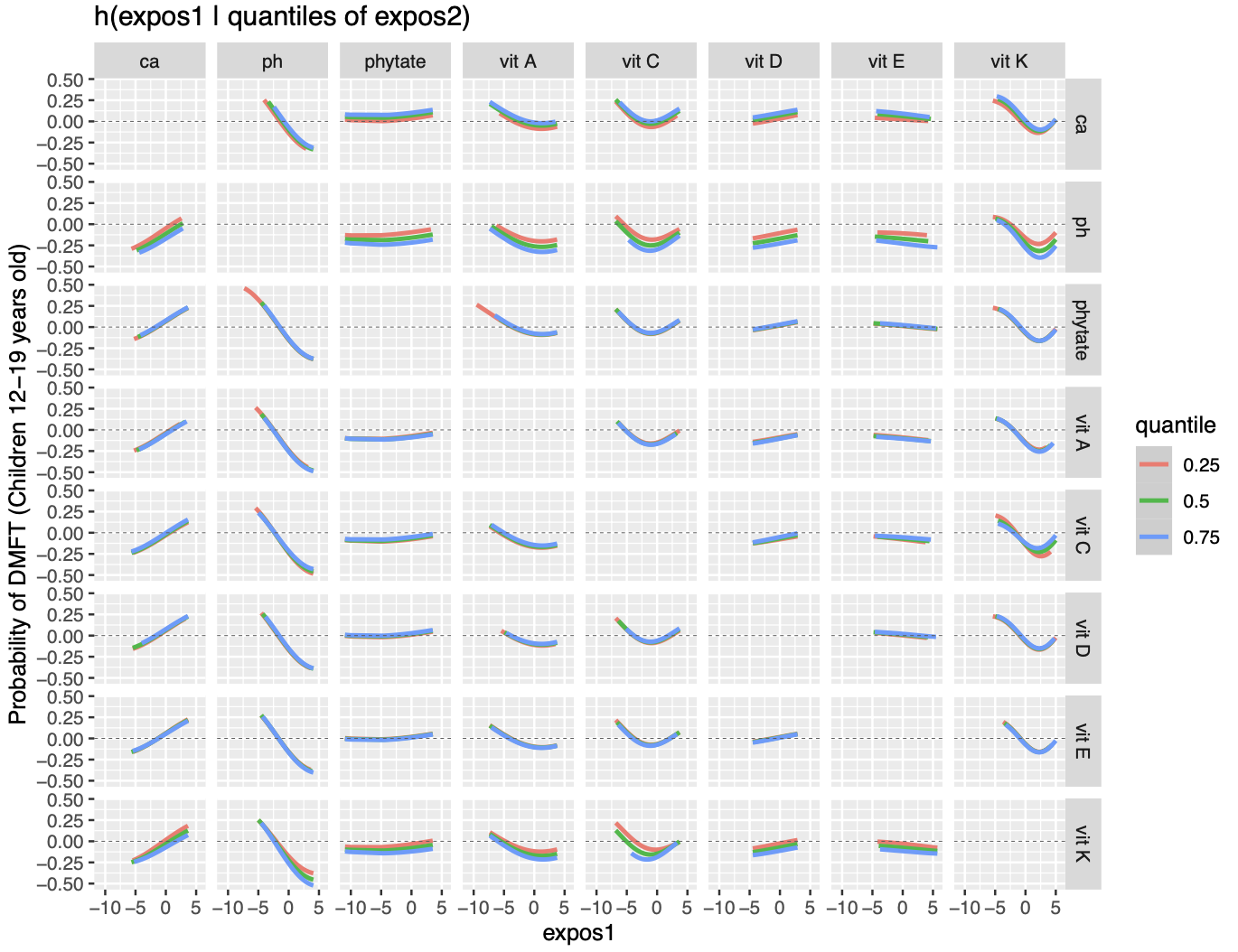 |
| --- |
| **Figure S4:** Bivariate concentration-response curves evaluating associations between each nutrient on the probability of DMFT relative to no DMFT in 12-19 years old (n=3,701). Figures illustrate the probit model estimates and 95% credible intervals for bivariate associations between all nutrients on probability of DMFT, while setting a second nutrient to its 25^th^, 50^th^, and 75^th^ percentile and all other nutrients to their median. Models were adjusted for age, sex, race and ethnicity, ratio of family income to poverty, total energy, and last dental visit. |

|  |
| --- |
| **Figure S5:** Single-exposure health effects plots examining the change in a single nutrient on DFT or DMFT, relative to participants with no DFT or DMFT. Figures illustrate the probit model estimates and 95% credible intervals for the change in a single nutrient on probability of DFT or DMFT, when all of the remaining exposures are fixed at their 25^th^ percentile as compared to when they are fixed at their 75^th^ percentile, when all other exposures are fixed at the 25^th^, 50^th^, and 75^th^ percentile. |
